# MicroRNA‐92a promotes vascular smooth muscle cell proliferation and migration through the ROCK/MLCK signalling pathway

**DOI:** 10.1111/jcmm.14274

**Published:** 2019-03-25

**Authors:** Jingyu Wang, Chenxu Zhang, Cai Li, Dandan Zhao, Shuyao Li, Le Ma, Ying Cui, Xiaoqing Wei, Ying Zhao, Ying Gao

**Affiliations:** ^1^ Department of Biochemistry and Molecular Biology, College of Basic Medical Sciences Dalian Medical University Dalian China; ^2^ College of Stomatology Dalian Medical University Dalian China; ^3^ Liaoning Provincial Key Lab of Medical Molecular Biology Dalian Medical University Dalian China

**Keywords:** miR‐92a, MLCK, ROCK, VSMC

## Abstract

To identify the interaction between known regulators of atherosclerosis, microRNA‐92a (miR‐92a), Rho‐associated coiled‐coil‐forming kinase (ROCK) and myosin light chain kinase (MLCK), we examined their expressions during proliferation and migration of platelet‐derived growth factor‐BB (PDGF‐BB)‐regulated vascular smooth muscle cells (VSMCs), both in vivo and in vitro. During the formation of atherosclerosis plaque in mice, a parallel increase in expression levels of MLCK and miR‐92a was observed while miR‐92a expression was reduced in ML‐7 (an inhibitor of MLCK) treated mice and in MLCK‐deficient VSMCs. In vitro results indicated that both MLCK and miR‐92a shared the same signalling pathway. Transfection of miR‐92a mimic partially restored the effect of MLCK's deficiency and antagonized the effect of Y27632 (an inhibitor of ROCK) on the down‐regulation of VSMCs activities. ML‐7 increased the expression of Kruppel‐like factor 4 (KLF4, a target of miR‐92a), and siRNA‐KLF4 increased VSMCs' activity level. Consistently, inhibition of either MLCK or ROCK enhanced the KLF4 expression. Moreover, we observed that ROCK/MLCK up‐regulated miR‐92a expression in VSMCs through signal transducer and activator of transcription 3 (STAT3) activation. In conclusion, the activation of ROCK/STAT3 and/or MLCK/STAT3 may up‐regulate miR‐92a expression, which subsequently inhibits KLF4 expression and promotes PDGF‐BB‐mediated proliferation and migration of VSMCs. This new downstream node in the ROCK/MLCK signalling pathway may offer a potential intervention target for treatment of atherosclerosis.

## 1 INTRODUCTION

Atherosclerosis (AS) and its associated clinical complications, such as myocardial infarction, stroke and peripheral artery disease, are the leading causes of morbidity and mortality worldwide. The pathogenesis of AS lesion formation results from a series of complicated cascade processes including endothelial dysfunction, neovascularization, vascular smooth muscle cells (VSMCs) proliferation and migration, apoptosis, matrix degradation, oxidative stress and inflammation. Although researches have identified some key signalling and molecular regulatory pathways involved in the initiation and progression of AS plaques, the pathophysiological mechanisms of AS have yet to be illuminated,^1^, ^2^ hence the prevention and treatment options for AS remain limited.

Endothelial cells (ECs) and VSMCs are the main cell types of within the vasculature and closely related in structure and function. ECs that cover the interior surface of blood play an important role in the regulation of the vascular tone by releasing vasoactive agents controlling VSMCs proliferation or migration.^3^, ^4^, ^5^ VSMCs that provide structural integrity to the vessel wall are fine‐tuned by adjacent ECs.^6^, ^7^ Aberrant proliferation and migration of VSMCs are the most studied key pathological processes in the initiation and development of AS.^8^, ^9^, ^10^, ^11^ Among the various factors associated with the development of AS, the high expression of platelet‐derived growth factor‐BB (PDGF‐BB) has been detected in nearly all cell types of the atherosclerotic aortic wall and in the infiltrating inflammatory cells.^12^ PDGF‐BB is a known potent mitogen and chemoattractant for VSMCs and is found in atherosclerotic lesions.^13^ Moreover, PDGF‐BB can activate Rho‐associated coiled‐coil‐forming kinase (ROCK) and myosin light chain kinase (MLCK),^14^, ^15^, ^16^, ^17^ both of which regulate phosphorylation of myosin light chain (MLC).^18^ The phosphorylation of MLC promotes the cell contraction and cell motility thereby leading changes in actin cytoskeleton.^18^, ^19^ The rearrangement of the actin cytoskeleton, in turn, may greatly influence inflammatory signalling.^20^ Therefore, blocking the PDGF‐BB‐induced ROCK/MLCK signalling pathway could potentially prevent the dysregulation of VSMCs, and consequently attenuate the progression of AS. Exploring the novel regulatory mechanisms of the PGDF‐BB signalling pathway could be of great scientific and therapeutic interest for AS.

MicroRNAs (miRNAs) are evolutionarily conserved, non‐coding small RNAs that can regulate gene expression at post‐transcriptional level, which means that one miRNA usually targets 3′‐untranslated regions of various mRNAs that are involved in different steps of one precise metabolic/signalling pathway. Therefore, changes in the levels of one key miRNA affect various steps of one pathway, which is thereby promoted or inhibited. This makes miRNAs potent future diagnostic and even therapeutic tools for personalized medicine.^21^ Recent findings have revealed a key role for miRNAs in the pathophysiological processes of cardiovascular disease, such as miR‐126, miR‐146, miR‐143/145 and others, have been identified as relevant mediators by modulating ECs and VSMCs function in angiogenesis, AS and in‐stent restenosis,^1^, ^5^ miR‐27a/b, miR‐33, miR‐122, miR‐144 or miR‐223 involved in lipid metabolism.^21^, ^22^ miR‐92a, a member of the miR‐17‐92 cluster, is highly expressed in ECs of blood vessel walls.^23^, ^24^, ^25^ The role of miR‐92a in the development of AS in vivo has been well‐documented.^23^, ^26^ Specifically, miR‐92a is highly expressed in athero‐prone areas of the aortic arch compared with athero‐resistant regions.^25^, ^27^ Up‐regulation of miR‐92a by oxidized low‐density lipoproteins (oxLDL), present in athero‐prone areas, enhances endothelial activation and atherosclerotic lesions' progression.^23^

As both the PDGF‐BB‐induced ROCK/MLCK signalling pathway and miR‐92a‐mediated post‐transcriptional effects are important aspects of the AS lesion formation, it is of interests to identify any potential connection between the two pathways. In this study, we observed that the expression levels of MLCK and miR‐92a were significantly increased in parallel during atherosclerotic plaque formation in mice. The inhibition of MLCK with its inhibitor ML‐7 reduced lipid deposition lesions, as well as miR‐92a expression, which was also found decrease in MLCK‐deficient VSMCs. We therefore highly speculated that MLCK could be involved in the regulation of miR‐92a in VSMCs, and tested our hypothesis in an in vitro model. Our attempt to unveil the complicated signalling network in AS lesion progression may provide clues in the development of novel clinical biomarkers or therapeutic targets.

## 2 MATERIALS AND METHODS

### 2.1 Reagents and antibodies

PDGF‐BB was purchased from PeproTech (100‐14B, Rocky Hill). GAPDH monoclonal antibodies (60004‐1‐Ig), KLF4 polyclonal antibodies (11880‐1‐AP) and STAT3 polyclonal antibodies (10253‐2‐AP) were purchased from Proteintech. Anti‐P‐STAT3 (Ser 727) Rabbit pAb (WL03346) were purchased from Wanleibio. MLCK monoclonal antibodies (ab76092), ML‐7 (ab120848), Y‐27632 dihydrochloride (ab120129) and 4,6‐diamidino‐2‐phenylindole (DAPI, ab104139) were obtained from Abcam Inc. The STAT3 inhibitor S3I‐201 was obtained from Selleck Chemicals (S1155). FITC‐conjugated α‐smooth muscle actin antibody (F3777) and Oil‐Red‐O solution (O1391) was purchased from Sigma‐Aldrich (St Louis, MO). Rhodamine conjugated goat anti‐rabbit IgG (H+L) (31670) was purchased from Thermo Scientific. Total cholesterol assay kit, Triglyceride assay kit, High‐density lipoprotein cholesterol assay kit, Low‐density lipoprotein cholesterol assay kit were purchased from Nanjing Jiancheng Bioengineering Institute (China).

### 2.2 Animal experiments

Homozygous male *ApoE* ^−^ *^/^* ^−^ mice (aged 6 weeks) on C57BL/6J background were originally purchased from Beijing Vital River Laboratory Animal Technology Co. Ltd. This study was conducted in accordance with the Guide for the Care and Use of Laboratory Animals published by the US National Institutes of Health (8th edition, 2011). The animal protocol was approved by the local research ethics review board of the Animal Ethics Committee of Dalian Medical University.

#### 2.2.1 AS model

Mice were randomly divided into control group (n = 50, standard chow fed) and high‐fat diet fed group (n = 50, high‐fat diet consisting of 78% common chow, 10% lard oil, 10% yolk powder, 1% cholesterol and 0.2% bile salt from pig). The mice from control and high‐fat fed group at 6, 9, 12, 15 and 18 weeks were dissected to examine the extent of AS.

#### 2.2.2 ML‐7 model

The AS mice were randomly divided into the untreated group (n = 16) and the ML‐7 treatment group (n = 16). All mice in both groups were fed with a high‐fat diet. ML‐7 group were treated with ML‐7 by injecting into the veins of the tails twice a week at a dose of 1 mg/kg from 6 weeks. The mice of untreated group were injected with the same volume of saline as a control. The body weights of all mice were measured at 6, 9, 12, 15 and 18 weeks. All mice at 18 weeks of age were dissected. Thoracic aorta was excised, snap frozen in liquid nitrogen and stored at −80°C for subsequent RT‐qPCR analysis.

### 2.3 Serum lipid analysis

Serum total cholesterol (T‐CHO), triglyceride (TG), High‐density lipoprotein (HDL) and low‐density lipoprotein (LDL) measurements were performed at 6, 9, 12, 15 and 18 weeks. Whole blood was obtained by retro‐orbital bleeding and centrifuged at 2000 *g* for 15 minutes at 4°C. Blood lipid analyses were measured using the commercial kits (Nanjing Jiancheng Bioengineering Institute, China) according to the manufacturers' instructions.

### 2.4 Haematoxylin and eosin (H&E) staining

Thoracic aortas were fixed with 4% paraformaldehyde, embedded in paraffin and sliced into 4 μm sections. The sections were baked at 70°C for 4 hours, dewaxed, hydrated in distilled water, stained with haematoxylin for 1 minute, differentiated in hydrochloric acid alcohol, blued in ammonia water, counter‐stained with eosin (7 seconds), dehydrated with ethanol, transparentized with xylene I and xylene II, and finally mounted in neutral gum.

### 2.5 Immunofluorescence staining

Paraffin‐sectioned slides from thoracic aortas tissues were used. First, the slides were deparaffinized and rehydrated by dimethylbenzene and ethanol. Antigen retrieval was performed by incubating slides in 0.01 mol/L citrate buffer (pH 6.0) at 95°C for 20 minutes. The samples were then blocked for 30 minutes, followed by an overnight incubation with the KLF4 antibodies (1:100). Next, the slides were rinsed with PBS and incubated with the Rhodamine conjugated goat anti‐rabbit IgG (H+L) (Thermo) for 30 minutes at 37°C. After being rinsed with PBS, the samples were incubated with FITC‐conjugated α‐smooth muscle actin antibody (1:100) for 40 minutes at 37°C. Finally, cell nuclei were counter‐stained with DAPI. Digital images were captured with a fluorescence microscope (BX‐51, TR32000; Olympus, Tokyo, Japan).

A7r5 or Gba cells were fixed in 4% formaldehyde, permeabilized with 0.1% Triton X‐100 for 10 minutes and blocked with 5% BSA for 20 minutes. The cells were incubated with the rabbit anti‐human KLF4 antibodies overnight at 4°C and washed, followed by a 1 hour incubation with the appropriate antibodies at room temperature, including Rhodamine conjugated goat anti‐rabbit IgG (H+L) (Thermo) and FITC‐conjugated F‐action α‐smooth muscle actin antibody (1:100). Nuclei were counter‐stained with DAPI and then observed under a fluorescence microscope.

### 2.6 Oil‐Red‐O staining

Thoracic aorta was washed with PBS and fixed with 78% methyl alcohol twice after the removal of aortic peripheral adipose tissue. The staining method is as follows: the fixed thoracic aorta samples were rinsed with 78% methyl alcohol for 5 minutes and stained in 0.5% Oil‐Red‐O solution for 1 hour. Thoracic aorta was differentiated in a 78% methyl alcohol solution for 5 minutes, and then was sliced longitudinally to expose the intimal surface. The stained thoracic aorta was spread on a black charpie for photographing using a digital camera under identical light conditions.

### 2.7 miRNA array analysis

MicroRNA array analysis from GbaSM‐4 and MLCK^−^/Gba total RNA was performed by Kangcheng Bio‐tech Inc (Shanghai, China).

### 2.8 Isolation and culture of primary rat aortic SMCs

Sprague‐Dawley rats (250‐300 g, from the animal experiment center of Dalian Medical University) were killed by diethyl ether. Thoracic aorta was dissected to remove adhering periadventitial tissue and the endothelium was denuded with a catheter. After removing the adventitial layer, the remaining medial layer was minced into small pieces for digestion with Collagenase I (Catalog No. 17100‐017, Gibco, Langley, OK) for 5 hours at 37°C. Then the small pieces of aorta were digested with 0.125% trypsin (Gibco) for 10 minutes at 37°C. Following the removal of digestion solution and re‐suspending in 10% FBS F‐12/DMEM medium, cells were gently transferred into culture dishes and incubated at 37°C. Every batch of VSMCs was tested by smooth muscle marker α‐smooth muscle actin staining to ensure the purity of primary VSMCs to be above 95%.

### 2.9 Cell culture

A7r5 is originally derived from the embryonic rat aorta and were purchased from the American Type Culture Collection (ATCC, Manassas, VA). GbaSM‐4 and MLCK^−^/Gba cell lines, originally derived from guinea pig basilar artery SMCs, were offered by Professor Kazuhiro Kohama from Gunma University. Human aortic SMCs (HASMCs) were purchased from Chi Scientific Inc (Catalog No. 7‐1562, Jiangsu, China). Both A7r5 and Gba were maintained in Dulbecco's modified Eagle's medium (Gibco) supplemented with 10% foetal bovine serum (Gibco) and 1% penicillin‐streptomycin. HASMCs were maintained in F‐12/DMEM (Hyclone, Logan, UT) supplemented with 10% foetal bovine serum (FBS500‐S; AusGenex, Australia) and 1% penicillin‐streptomycin. All cells were maintained at 37°C in 5% CO_2_.

### 2.10 RNA extraction and quantitative real‐time PCR (qRT‐PCR)

Total RNA from cells or tissues was extracted with Trizol reagent (Ambion, Life Technologies) and reverse transcribed into cDNA using the TransScript One‐Step gDNA Removal and cDNA Synthesis SuperMix (TransGen Biotech, Beijing) or TransScript miRNA First‐Strand cDNA Synthesis SuperMix (TransGen Biotech) according to the manufacturers' instructions. The expression levels of different genes or miRNA were quantified by RT‐qPCR using the TransStartTop Green qPCR SuperMix Kit (TransGen Biotech). Real‐time quantitative PCR was performed by the Agilent StrataGene Mx3000P Multiplex Quantitative PCR System (Stratagene, Agilent). The results were analysed by StrataGene Mx3000P software with GAPDH (for mRNA) or U6 (for miRNA) as an internal control. The primers were listed in Table 1.

**Table 1:** Primers used in qRT‐PCR

Gene	Forward sequence	Reverse sequence	PCR size
*miR‐92a*	TATTGCACTTGTCCCGGCCTG	TTTTTTTTTTTTTTTTTTTTTTT	89
*U6*	CTCGCTTCGGCAGCACA	AACGCTTCACGAATTTGCGT	94
Mouse *GAPDH*	TGTGTCCGTCGTGGATCTG	TTGCTGTTGAAGTCGCAGGAG	150
Human* GAPDH*	GAGTCAACGGATTTGGTCGT	GACAAGCTTCCCGTTCTCAG	185
Rat *GAPDH*	GCAAGTTCAACGGCACAG	GCCAGTAGACTCCACGACAT	140
Mouse *MLCK*	TGGGGGACGTGAAACTGTTTG	GGGGCAGAATGAAAGCTGG	114
Human *MLCK*	GTGACATGGCACAGAAACGG	CCAAGCTGCTTCGCAAAACT	206
Rat *ROCK*	AACTTTTGGACCTTTCGGATTC	TTGCTGCTCACCACAACATACT	166
Human *ROCK*	GGTAAGGCATAAATCCAC	TTCAGGCACATCATAGTT	241

### 2.11 Western blot analysis

Cells were lysed in RIPA buffer (Beyotime, China). Equal amounts of proteins were separated by SDS‐PAGE and electrotransferred to polyvinylidene difluoride membranes (Bio‐Rad). After blocking in 5% skim milk powder, the membranes were incubated with the following primary antibodies: GAPDH (1:10 000), MLCK (1:2000), KLF4 (1:1500), phospho‐S727 STAT3 (1:1000), STAT3 (1:500). Immuno‐reactive proteins were visualized with enhanced chemiluminescence (ECL) detection system (Advansta, Menlo Park, CA). GAPDH was used as a control.

### 2.12 CCK‐8 assay

Cells were transferred into 96‐well plates at 24 hours after transfection. The medium was changed to serum‐free medium with or without 10 ng/mL PDGF‐BB for 24 hours. Ten‐microlitre of CCK‐8 solution (TransGen Biotech) was added to each well and absorbance was measured at 450 nm. The results are presented as a percentage of cell proliferation (the optical density [OD] of the experiment samples/control group).

### 2.13 Wound‐healing assay

Cells were seeded in 6‐well plates and cultured until 90% confluence after 24 hours of transfection. Then cell layer were scratched with a 200‐μL sterile pipette tip. The cells were washed three times with serum‐free media to remove detached cells. After 10 hours incubation, cell migration was viewed and photographed using an optical microscope (BX‐51, TR32000; Olympus).

### 2.14 Boyden chamber assay

Cell migration was performed with a Boyden Chamber with polycarbonate filters with 8 µm pore size (Neuro Probe, Inc, Gaithersburg, MD) coated with collagen. A7r5 or Gba cells with different treatments were re‐suspended in serum‐free DMEM medium containing 0.4% BSA and 1 × 10^4^ cells were placed into the upper chamber. The lower chamber was filled with serum‐free medium containing 0.4% BSA with or without PDGF‐BB (10 ng/mL). After 5 hours incubation, the cells that penetrated and attached to the bottom of the filter were fixed in methanol and stained with Giemsa solution at room temperature for 20 minutes. The cells were observed using microscopy and the cell counts from randomly selected fields were recorded for each well.

### 2.15 Cell transfections

miR‐92a mimic, miR‐92a inhibitor, siRNA‐KLF4, siRNA‐ROCK, siRNA‐STAT3 and negative control oligonucleotide were purchased from Genepharma, Shanghai, China. miR‐92a mimic or miR‐92a inhibitor was added to media at a final concentration of 50 nmol/L. Transfections were performed with Lipofectamine 2000 (Invitrogen, Carlsbad, CA) according to the manufacturer's instructions. Transfected cells were used at 24 or 48 hours.

### 2.16 Statistical analysis

Statistical analysis was performed with GraphPad Prism 6 and presented as mean ± SD from at least three independent experiments. Student's *t* test method was utilized for comparison between two groups while one‐way anova followed by Tukey's test was used for data comparison in multiple group comparison. *P < *0.05 was considered as statistically significant. We used Kolmogorov‐Smirnov test to check whether each set of data are all normal distribution or not before using Student's *t* test for statistical analysis.

## 3 RESULTS

### 3.1 MLCK and miR‐92a are involved in the formation of AS plaque

**Figure 1:**
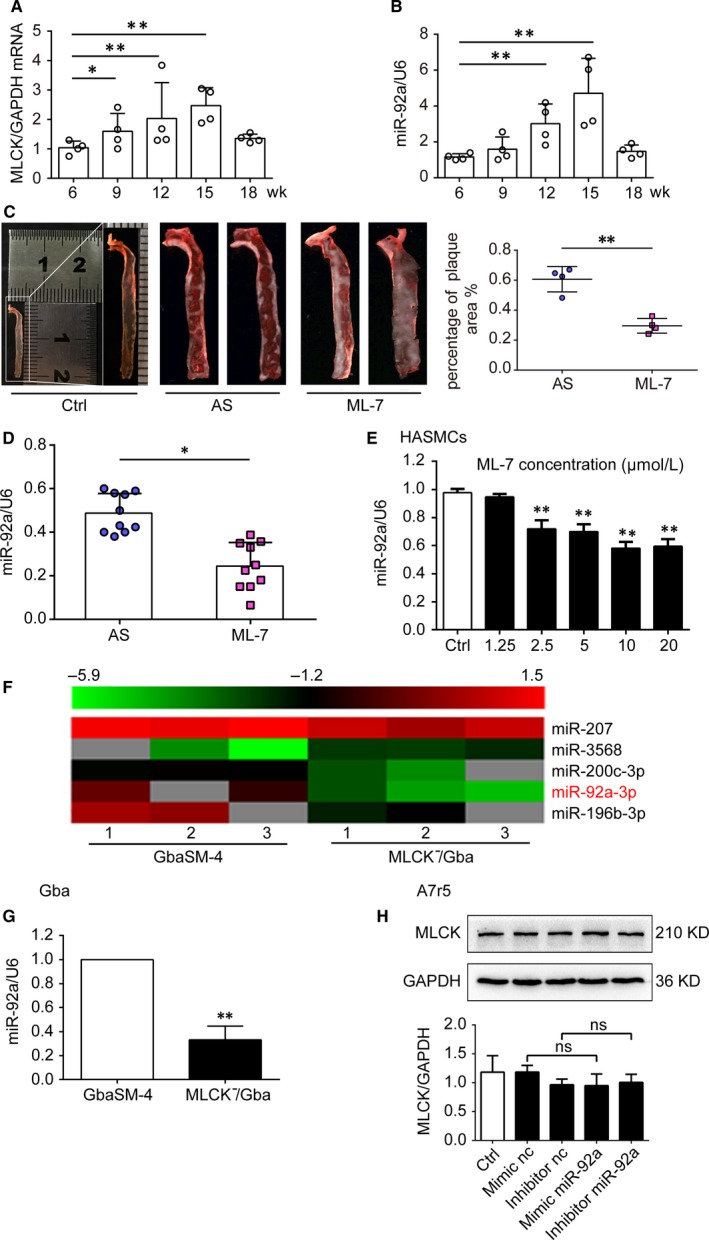
Expression of myosin light chain kinase (MLCK) and microRNA‐92a (miR‐92a) during the AS formation. A and B, MLCK mRNA (A) and miR‐92a (B) expression were assessed by RT‐qPCR in isolated aortic vessels from mice (n = 4). C, Representative photographs of Oil Red O stained aortic wall at 18 wk (n = 4). Results of statistical analyses are shown on the right. D, miR‐92a expression were assessed by RT‐qPCR in isolated aortic vessels from ML‐7 model group mice (n = 10). E, miR‐92a expression levels were assessed by RT‐qPCR in ML‐7 treated HASMCs cells. F, Hierarchical clustering analysis of miRNA expression in GbaSM‐4 and MLCK^-^/Gba cells (n = 3). G, miR‐ 92a expression levels were measured by RT‐qPCR. H, The protein level of MLCK was verified by Western blot analyses. Results of statistical analyses are shown on the below. GAPDH was used as a loading control. Data are presented as mean ± SD from at least three independent experiments. *P < 0.05, **P < 0.01

The development of AS is a dynamic process in which key signalling and molecular regulatory pathways are involved in the initiation and progression of AS plaques.^28^ We generated an AS model using *ApoE* ^−^ *^/^* ^−^ mouse^29^ and monitored the plasma lipid levels and the changes of aortic walls in mice during AS formation every 3 weeks from 6 weeks of age. The levels of triglyceride (TG), total cholesterol (T‐CHO) and low‐density lipoprotein (LDL) were significantly increased at 9 weeks, and reached its peak threshold at 9 and 12 weeks. HDL significantly decreased and reached its lowest point at 15 weeks (Figure S1A‐D). Histologically, the thickening of blood vessel wall was observed at 15 weeks whereas clear plaque was generated at 18 weeks (Figure S1E). During the time‐course of AS formation, the expression levels of MLCK and miR‐92a were significantly increased at 9 and 12 weeks, and both reached its peak at 15 weeks (*P* < 0.01, Figure 1A,B), in line with the thickening of blood vessel wall, suggesting that both MLCK and miR‐92a are involved in the formation of AS.

### 3.2 Inhibition of MLCK activity down‐regulated miR‐92a expression and reduced lipid deposition lesions in AS mice

This parallel increase in both the expression of MLCK and miR‐92a indicates that there is a possible connection between MLCK and miR‐92a in AS formation process. To verify this possibility, we performed a series of experimental procedures, both in vivo and in vitro*. *We first inhibited MLCK activity with its specific inhibitor ML‐7. ML‐7 was injected to AS mice via tail vein twice per week. No significant difference of body weight between ML‐7‐treated and untreated mice were found (Figure S1F). However, after ML‐7 treatment, the level of LDL was significantly decreased compared with untreated mice (*P < *0.05, Figure S1G), and aortic lipid deposition lesions were significantly reduced as well (*P < *0.05, Figure 1C). Strikingly, miR‐92a expression in aortic wall also declined significantly at the same time (*P < *0.05, Figure 1D), suggesting that miR‐92a may share the same pathway with MLCK. In line with these in vivo data, the expression of miR‐92a was also found to be down‐regulated in ML‐7 treated human aortic smooth muscle cells (HASMCs) (*P < *0.01, Figure 1E).

### 3.3 Absence of MLCK gene in VSMCs leads to reduced expression of miR‐92a

To further explore the connection between MLCK and miR‐92a, we examined miR‐92a expression in a guinea pig basilar artery smooth muscle cell line GbaSM‐4 (wild‐type) and its MLCK‐deficient format (MLCK^−^/Gba). The expression level of MLCK in MLCK^−^/Gba was significantly down‐regulated when compared to GbaSM‐4 (*P < *0.01, Figure S2). miR‐92a was identified as the most dysregulated miRNA in GbaSM‐4 vs MLCK^−^/Gba by Volcano Plot analysis (Figure 1F). Next, we validated the microarray data by RT‐qPCR. The expression level of miR‐92a was significantly lower in MLCK^−^/Gba compared to that of wild‐type GbaSM‐4 (*P < *0.01, Figure 1G). In contrast, after up‐ or down‐regulating the miR‐92a expression in VSMCs with miR‐92a mimic or miR‐92a inhibitor, respectively, MLCK expression remained at similar levels (Figure 1H). These findings suggest that MLCK could be the upstream modulator of miR‐92a in VSMCs.

### 3.4 MLCK and miR‐92a both regulate the migration and proliferation of VSMCs

**Figure 2:**
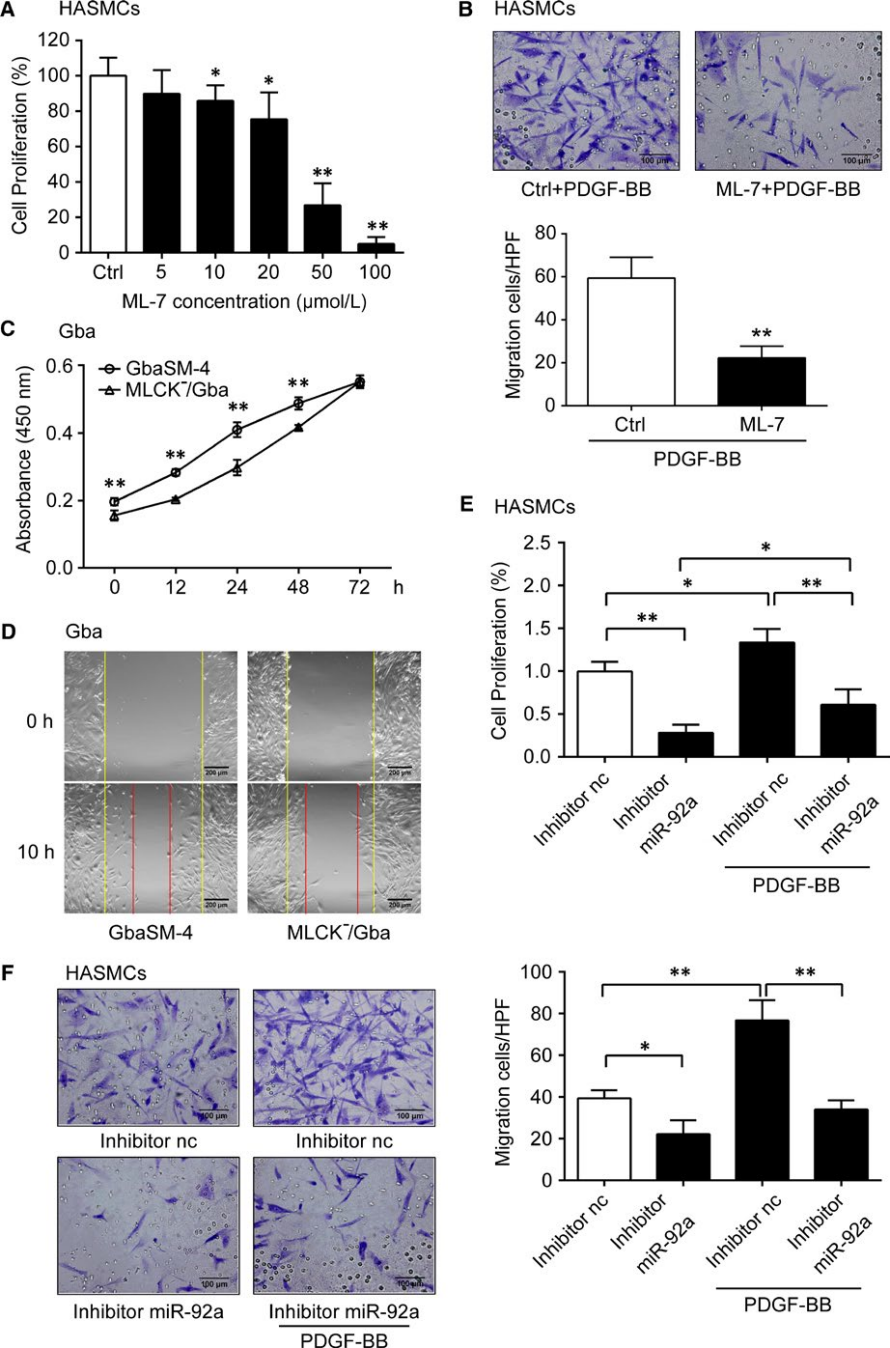
Effects of myosin light chain kinase (MLCK) and microRNA‐92a (miR‐92a) on the migration and proliferation of vascular smooth muscle cells (VSMCs). A, The cell proliferative ability was measured by CCK8 in ML‐7 (5‐100 μmol/L) treated HASMCs for 36 h. B, HASMCs were treated with 10 μmol/L ML‐7 or not. The cells migration was assessed by the Boyden chamber assay with 10 ng/mL PDGF‐BB. Migrated cells in each high‐power field (HPF, 400×) were quantitated and the results are shown below (n = 5). C and D, The cells proliferation and migration of GbaSM‐4 and MLCK^-^/Gba cells were measured by CCK8 (C) and wound healing assay (D) respectively. Bars, 200 μm. E, The proliferation rates of HASMCs were measured by CCK8 assay with or without 10 ng/mL PDGF‐BB after transfected. F, The cell migration was assessed by Boyden Chamber assay with or without 10 ng/mL PDGF‐BB after transfected. Migrated cells in each high‐power field (HPF, 400×) were quantitated and the results are shown on the right (n = 5). Data are presented as mean ± SD from at least three independent experiments. *P < 0.05, **P < 0.01

It is well‐known that dysregulated proliferation and migration of VSMCs in response to environmental stimuli play key roles in the development of AS.^8^, ^9^ PDGF‐BB was originally identified as the platelet and serum mitogen for regulating proliferation and migration of VSMCs.^12^, ^30^ To further determine the relationship between MLCK and miR‐92a, we studied their roles in proliferation and migration of PDGF‐BB stimulated VSMCs. The expression of MLCK and miR‐92a was elevated by the stimulation of VSMCs with PDGF‐BB (Figure S3A‐C). The role of MLCK was then studied in this system by inhibiting either its function or expression. The proliferation of HASMCs was inhibited by ML‐7 at a concentration ranging from 10 to 100 μmol/L in a dose‐dependent manner (Figure 2A). Meanwhile, ML‐7 also inhibited the migration of HASMCs (*P < *0.01, Figure 2B). Compared to wild‐type GbaSM‐4 cells, the proliferation and migration of MLCK^−^/Gba cells were significantly decreased (Figure 2C,D). To study the role of miR‐92a in this system, we altered miR‐92a expression with miR‐92a inhibitor fragments in HASMCs (*P < *0.01, Figure S4A). The results indicated that miR‐92a inhibitor also suppressed the proliferation and migration of HASMCs regardless of the PDGF‐BB presence (Figure 2E,F).

### 3.5 miR‐92a mimic partially restored the MLCK deficiency induced impairment of proliferation and migration of VSMCs

**Figure 3:**
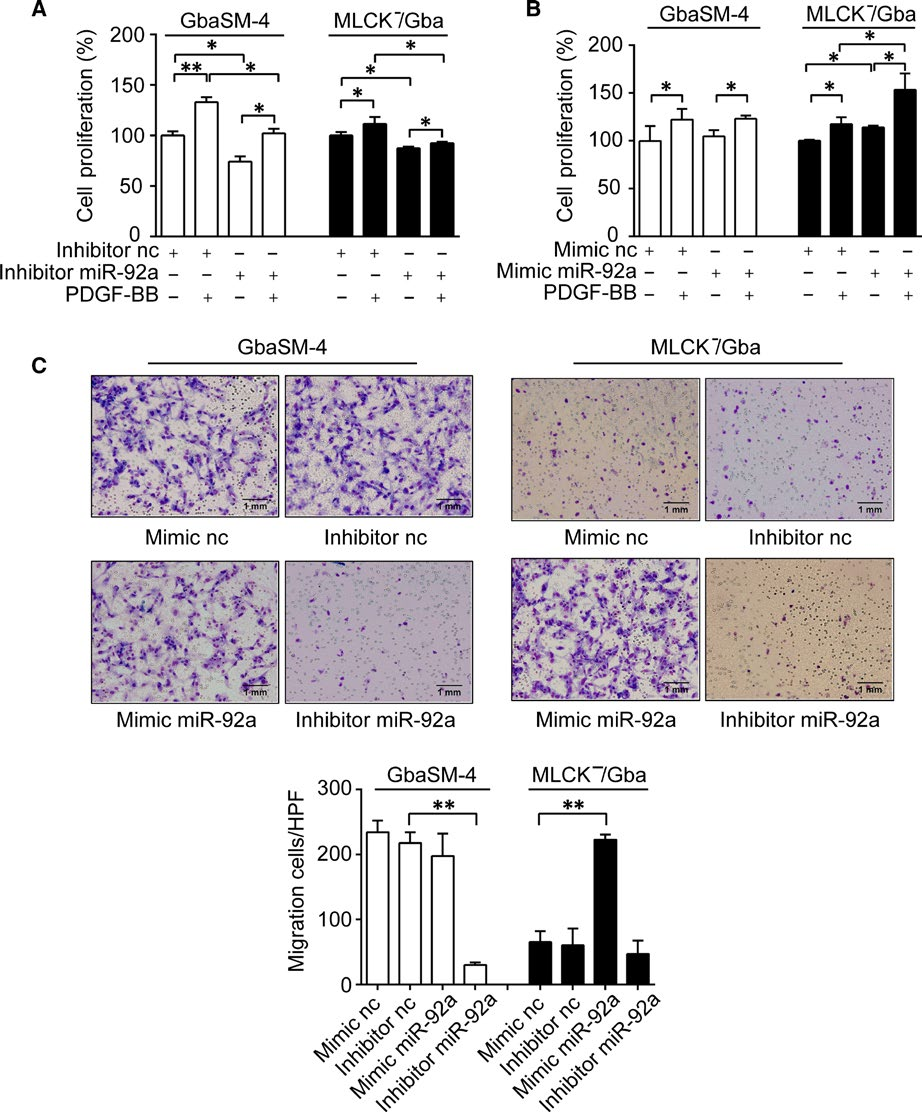
Effects of up‐ or down‐regulation of microRNA‐92a (miR‐92a) on proliferation and migration in GbaSM‐4 and MLCK^-^/Gba cells. A and B, The proliferation rates of GbaSM‐4 and MLCK^-^/Gba cells were measured by CCK8 assay with or without 10 ng/mL PDGF‐BB after transfected with miR‐92a inhibitor (A) or miR‐92a mimic (B). C, The cell migration was assessed by Boyden Chamber assay with 10 ng/mL PDGF‐BB after transfected. Migrated cells in each high power field (HPF, 100×) were quantitated and the results are shown on the right. Data are presented as mean ± SD from at least three independent experiments. *P < 0.05, **P < 0.01

To further explore the connection between MLCK and miR‐92a in VSMCs, we transfected miR‐92a inhibitor or miR‐92a mimic into GbaSM‐4 and MLCK^−^/Gba cells separately (Figure S4B,C). In GbaSM‐4 cells, PDGF‐BB obviously promoted the proliferation of VSMCs, whereas miR‐92a inhibitor effectively inhibited the PDGF‐BB induced proliferation (*P < *0.05, Figure 3A). miR‐92a mimic, however, did not further enhance the PDGF‐BB‐induced proliferative effects (Figure 3B). In MLCK^−^/Gba cells, PDGF‐BB also promoted the proliferation of cells (*P < *0.05), although its effect was significantly weaker than the effects observed in GbaSM‐4 cell. Meanwhile, the inhibitory effect of miR‐92a inhibitor on MLCK^−^/Gba cells proliferation (*P < *0.05) was consistent with the GbaSM‐4 cells (Figure 3A). Notably, miR‐92a mimic significantly promoted PDGF‐BB‐induced proliferation in MLCK^−^/Gba cells (*P < *0.05, Figure 3B). Similar result was observed in the migration experiments as well (Figure 3C). Transfection of miR‐92a inhibitor almost completely abolished the migration of GbaSM‐4 cells through Boyden Chamber assay (*P < *0.01) while miR‐92a mimic transfection dramatically promoted the migration of MLCK^−^/Gba cells (*P < *0.01, Figure 3C). These results indicated that both MLCK and miR‐92a could be components of the same signalling pathway regulating proliferation and migration of VSMCs with the MLCK likely being an upstream regulator of miR‐92a.

### 3.6 Changes in miR‐92a expression alter the inhibition of ROCK induced down‐regulation of cell proliferation and migration

**Figure 4:**
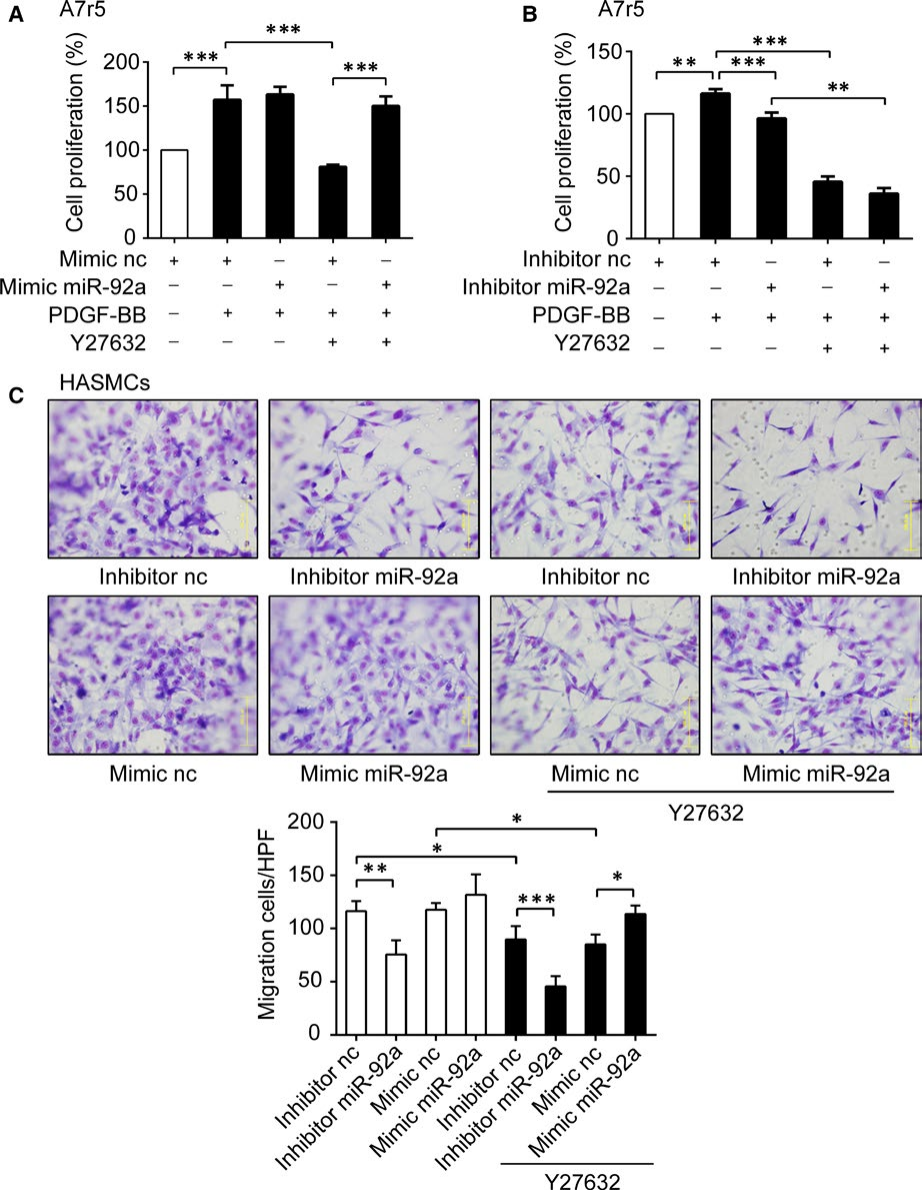
Effects of up‐ or down‐regulation of microRNA‐92a (miR‐92a) on proliferation and migration in Y27632 pretreated vascular smooth muscle cells (VSMCs). A and B, The proliferation rates of Y27632‐pretreated A7r5 cells were measured by CCK8 after transfected with miR‐92a mimic (A) or miR‐92a inhibitor (B). C, Y27632‐pretreated‐HASMCs were transfected with miR‐92a inhibitor or miR‐92a mimic. The cell migration was assessed by Boyden Chamber assay with 10 ng/mL PDGF‐BB stimulation. Migrated cells in each high‐power field (HPF, 400×) were quantitated and the results are shown on the right. Data are presented as mean ± SD from at least three independent experiments. *P < 0.05, **P < 0.01, ***P < 0.001

It is known that PDGF‐BB induced VSMCs dysfunction is crucially dependent upon the ROCK/MLCK signalling pathway.^14^, ^15^, ^16^ As our results identified MLCK as an upstream regulator of miR‐92a, it is of interest to find out whether ROCK possesses similar role as MLCK in regulating miR‐92a expression in VSMCs. We found that transfection with miR‐92a mimic or miR‐92a inhibitor did not alter the ROCK expression at the transcriptional level in A7r5 cells (Figure S5A). The inhibition of ROCK activity with its inhibitor Y27632 led to decreased expression of miR‐92a in A7r5 cells (*P < *0.01, Figure S5B) with subsequent reduction in A7r5 cell proliferation (Figure 4A,B) and reduced migration of HASMCs (Figure 4C). However, transfection of miR‐92a mimic showed no effect on proliferation of PDGF‐BB treated A7r5 cells, although it significantly antagonized the inhibitory effect of Y27632 on proliferation of A7r5 cells (*P < *0.001, Figure 4A) and on migration of HASMCs (*P < *0.05, Figure 4C). Transfection of miR‐92a inhibitor did not further decrease the proliferation of Y27632‐treated A7r5 cells (Figure 4B). Nevertheless, the transfection of miR‐92a inhibitor did significantly further inhibit the migration of HASMCs treated with Y27632 (*P < *0.001, Figure 4C).

### 3.7 ML‐7 increased the KLF4 expression of aortic SMCs in mice, and siRNA‐KLF4 increased the proliferation and migration of VSMCs

**Figure 5:**
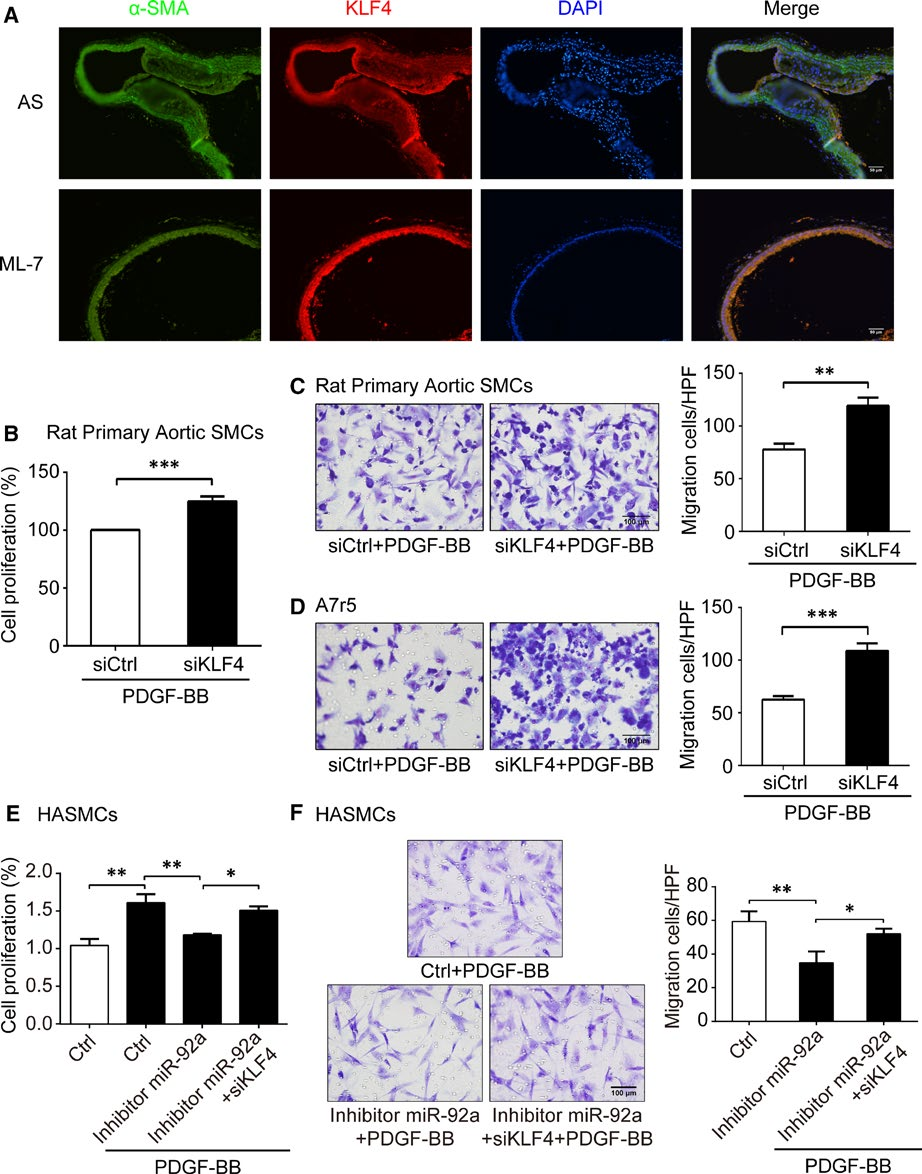
Expression of KLF4 in blood vessel walls of AS mice and effects of siRNA‐KLF4 (siKLF4) on vascular smooth muscle cells (VSMCs) function. A, Immunofluorescence staining for KLF4 (red) and α‐SMA (green) in AS mice and ML‐7 treatment mice. Nuclei were stained with DAPI (blue). Bars, 50 μm. B, The proliferation of siKLF4‐transfected Rat Primary Aortic SMCs were measured by CCK8 with 10 ng/mL PDGF‐BB stimulation. C and D, The migration of siKLF4‐transfected Rat Primary Aortic SMCs (C) and A7r5 (D) were measured by Boyden Chamber assay with 10 ng/mL PDGF‐BB stimulation. Migrated cells in each high‐power field (HPF, 400×) were quantitated and the results are shown on the right. E and F, The cells proliferation and migration of HASMCs were measured by CCK8 (E) and Boyden Chamber assay (F) respectively. Migrated cells in each high‐power field (HPF, 400×) were quantitated and the results are shown on the right. Data are presented as mean ± SD from three independent experiments. *P < 0.05, **P < 0.01, ***P < 0.001

Studies have shown Kruppel‐like factor 2 (KLF2), KLF4 and suppressor of cytokine signalling 5 (SOCS5) to be the targets of miR‐92a, which regulates gene networks involved in the process of AS.^23^, ^24^, ^25^ KLF4, a key suppressor of VSMCs proliferation, was increased in AS lesions of C/EBP homologous protein (CHOP) deletion mice, and silencing *KLF4* in CHOP‐deficient VSMCs restored proliferation.^31^ To better understand the role of miR‐92a, we examined KLF4 protein expression in the blood vessel walls from AS mice and ML‐7‐treated mice by immunofluorescence staining. We founded that, consistent with the result from oil‐red staining (Figure 1C), there was no significant plaque formation in the aorta of ML‐7‐treated mice. Interestingly, the KLF4 protein staining was enhanced with ML‐7‐treated mice compared to that of AS mice, and the KLF4‐positive cells were mainly distributed in the SMCs from the blood vessel walls (Figure 5A). On the other hand, in our in vitro system, transfection of siRNA‐KLF4 increased the proliferation (*P < *0.001) and migration (*P < *0.01) of rat primary aortic SMCs (Figure 5B,C). Migration of A7r5 cells was also significantly increased by blocking KLF4 expression with siRNA (*P < *0.001, Figure 5D). siRNA‐KLF4 could partially rescue the effects of inhibitor miR‐92a on PDGF‐BB‐mediated proliferation and migration of HASMCs (Figure 5E,F).

### 3.8 Expression of KLF4 was increased in MLCK^−^ and ROCK^−^ VSMCs

**Figure 6:**
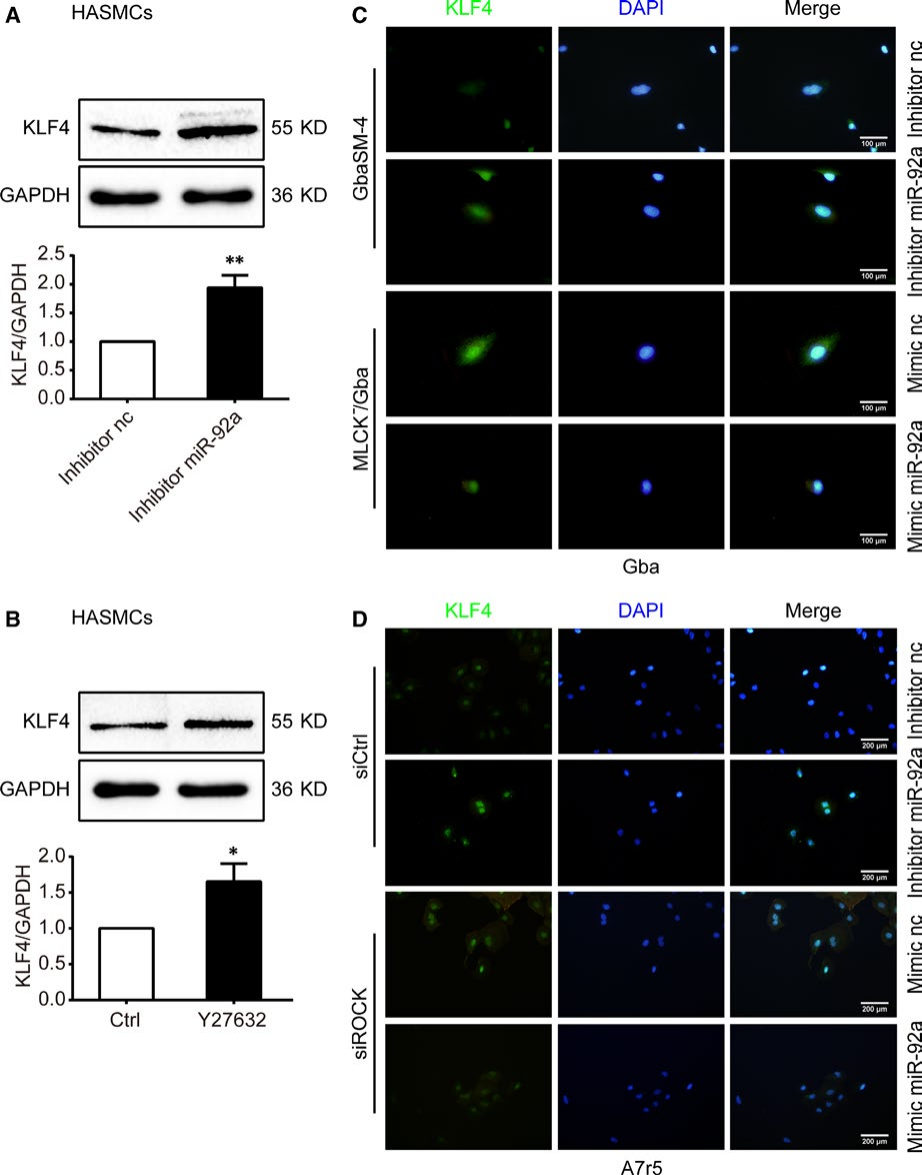
Effects of microRNA‐92a (miR‐92a) on KLF4 expression in vascular smooth muscle cells (VSMCs). A and B, The protein level of KLF4 were measured by Western blot analyses in HASMCs. Results of statistical analyses are shown below. C and D, Immunofluorescent staining for KLF4 (green) in Gba (C) or A7r5 (D) cells treated with 10 ng/ml PDGF‐BB after transfected. Nuclei demonstrated by DAPI (blue). Scale bars: 100 μm (C) and 200 μm (D). nc, negative control. Data are presented as mean ± SD from three independent experiments. *P < 0.05, **P < 0.01

Next, we examined the effect of MLCK and ROCK on KLF4 expression. Western blot analyses showed that both miR‐92a inhibitor transfection (*P < *0.01) and Y27632 treatment (*P < *0.05) increased KLF4 expression (Figure 6A,B). In MLCK^−^/Gba cells and siRNA‐ROCK transfected A7r5 cells, immunofluorescence labeling results showed that KLF4 protein staining was enhanced (Figure 6C,D).

### 3.9 ROCK/MLCK up‐regulated miR‐92a expression in VSMCs through STAT3 activation

**Figure 7:**
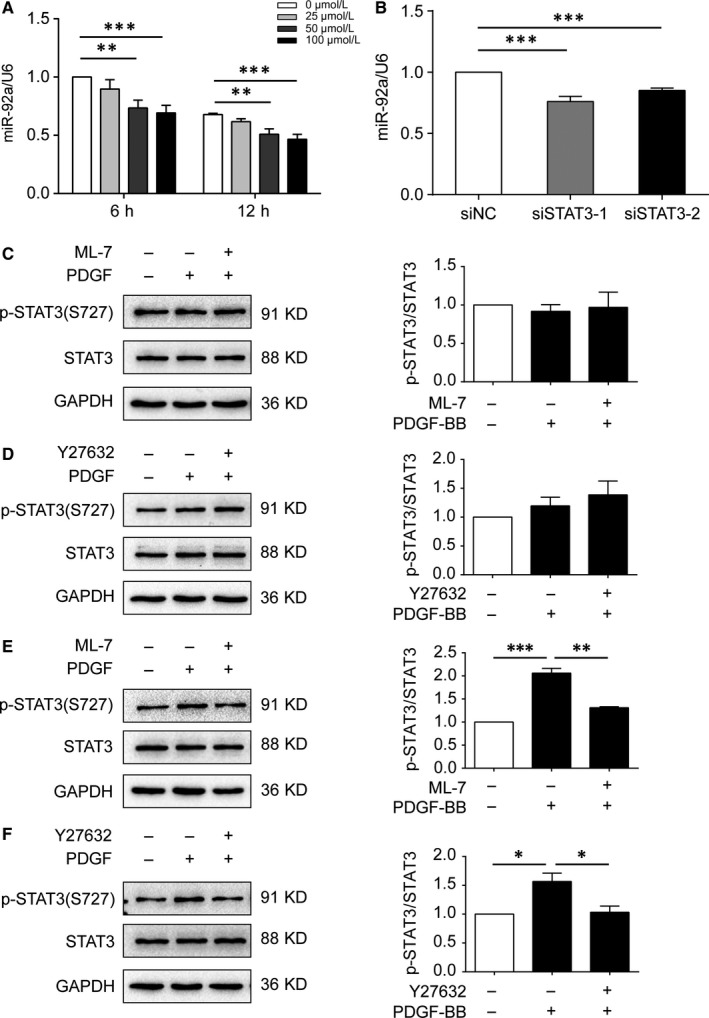
Effects of down‐regulation of STAT3 on microRNA‐92a (miR‐92a) and effects of inhibitors of myosin light chain kinase (MLCK) or ROCK on STAT3 in HASMCs. A and B, miR‐92a expression levels were assessed by RT‐qPCR in S3I‐201 treated HASMCs for the times indicated (A) or siRNA‐STAT3 (siSTAT3) transfected HASMCs (B). C‐F, HASMCs were pretreated with ML7 (10 μmol/L) or Y27632 (10 μmol/L) for 1 h, followed by 10 ng/mL PDGF‐BB for 1 h (C and D) or 12 h (E and F). The protein level of phospho‐S727 STAT3 and STAT3 were measured by Western blot analyses in HASMCs. Results of statistical analyses are shown below. Data are presented as mean ± SD from three independent experiments. *P < 0.05, **P < 0.01,***P < 0.001

Given the noticeable data that ROCK/MLCK up‐regulated miR‐92a expression in VSMCs, we sought to further explore the mechanistic relationship between ROCK/MLCK and miR‐92a expression. Because the promoter region of the miR‐92a gene contains a conserved binding site for Stats3,^32^ we postulated that activation of ROCK/STAT3 or MLCK/STAT3 might be a potential mechanism for miR‐92a up‐regulation in VSMCs. Indeed, the treatment of HASMCs with the S3I‐201 (an inhibitor of STAT3) or siRNA‐STAT3 down‐regulated the expression of miR‐92a (Figure 7A,B). Similarly, pretreatment of HASMCs with ML‐7 (10 μmol/L) or Y27632 (10 μmol/L) also decreased the expression of phospho‐S727 STAT3 in PDGF‐BB‐stimulated‐HASMCs at 12 hours (Figure 7E,F). But these changes were not observed at 1 hour (Figure 7C,D). These findings suggest an important role of ROCK/STAT3 or MLCK/STAT3 signalling for up‐regulation of miR‐92a expression in VSMCs.

## 4 DISCUSSION

MicroRNAs have been proven to regulate a wide range of biological processes, some of which are associated with AS.^33^ Loyer *et al* clearly demonstrated that in vivo inhibition of miR‐92a in *ldlr* ^−^ *^/^* ^−^ mice restricted the development of AS.^23^ We found that miR‐92a expression gradually increased with the formation of atherosclerotic plaques in *ApoE* ^−^ *^/^* ^−^ mice. MLCK induces contraction of the peri‐junctional apical acto‐myosin ring in response to phosphorylation of MLC. Abnormal expression of MLCK has been observed in respiratory diseases, pancreatitis, cardiovascular diseases, cancers and inflammatory bowel diseases.^17^ Cheng et al indicated that ML‐7, a MLCK inhibitor, may inhibit the AS rabbit's plaque formation by improving vascular endothelial dysfunction.^34^ In line with previous reports, we observed that ML‐7 significantly inhibited LDL level and aortic lipid deposition lesions of mice. These animal model studies have shown separately that miR‐92a or MLCK plays a critical role in AS plaque formation. In this study, we further discovered that the miR‐92a expression of the aorta was reduced in *ApoE* ^−^ *^/^* ^−^ mice treated with ML‐7, suggesting a possible link between MLCK and miR‐92a regarding their roles in the development of AS.

The development of the vascular architecture involves the strict association between ECs and mural cells, including VSMCs and pericytes. Under physiological conditions, the communication between these cell types leads to the maturation and stabilization of the vessel.^35^, ^36^ Vascular endothelial activation and inflammation are earlier clinical events which have been causally linked to VSMCs accumulation in several vascular disease models.^7^, ^37^ These processes involve the action of different miRNAs.^1^, ^5^, ^22^ Climent and colleagues elegantly demonstrated that VSMCs communicate with ECs via miR‐143 and miR‐145: cell‐to‐cell VSMCs/ECs contacts induce the activation of miR‐143/145 transcription in VSMCs, promoting the transfer of these miRNAs to the endothelium. In particular, VSMCs can deliver miR‐143/145 to ECs via fine intercellular tubes, named membrane nanotubes or tunnelling nanotubes.^38^ Previous studies on mechanism of AS involving miR‐92a or MLCK are mainly focused on endothelial dysfunction. miR‐92a inhibition in mice and large animal models has been shown to decrease plaque size of AS and protect against ischemia/reperfusion injury by reducing endothelial inflammatory activation.^22^, ^23^, ^24^, ^25^, ^26^, ^39^, ^40^ Inhibition of MLCK with ML‐7 improves vascular endothelial dysfunction via tight junction regulation in a rabbit model of AS.^17^, ^34^ However, only scattered evidence suggested a role of miR‐92a or MLCK on the biological function of VSMCs. VSMCs are traditionally associated with the fibrous cap. Aberrant proliferation and migration of VSMCs were the key pathological processes in the genesis and development of AS.^10^, ^11^ Growth factors and cytokines induce VSMCs proliferation and migration from the tunica media into the intima, ultimately resulting in fibrous cap formation of the plaque.^41^ Overexpression miR‐92a is known to inhibit hydrogen peroxide‐induced VSMCs migration and apoptosis.^42^, ^43^ ML‐9, a specific inhibitor of MLCK, inhibits PDGF‐BB and LPA‐inducted SMC migration.^9^ In this study, we focused on the effect of MLCK and miR‐92a on the function of VSMCs, and the regulatory relationship between them.

By miRNA microarray, we found miR‐92a expression reduced in VSMCs in the absence of *MLCK *gene. The same result was verified in ML‐7‐treated VSMCs, while the changes of miR‐92a gene level showed no effect on the expression of MLCK in VSMCs. These results strongly suggested that MLCK could be an upstream molecule of miR‐92a. We hypothesized that MLCK might be involved in the development of AS by regulating miR‐92a and its target genes, which affect the function of VSMCs in turn. However, our hypothesis seems to be inconsistent with the limited number of previous studies on the relationship between MLCK and miRNAs in which the up‐regulation of miR‐347a, miR‐155a, miR‐520c‐3p and miR‐1290 reduced MLCK expression in various tissues.^33^, ^44^ To test our hypothesis, we observed the effects of MLCK and miR‐92a on the function of VSMCs and the regulatory relationship between them. Our results showed that the proliferation and migration of MLCK^−^/Gba and ML‐7‐treated HASMCs were decreased, and miR‐92a inhibitor also inhibited VSMCs proliferation and migration. It is noteworthy that miR‐92a mimic partially rescued the effect of MLCK absence on down‐regulation of VSMCs proliferation and migration. These results showed for the first time that MLCK promoted the proliferation and migration of VSMCs through the regulation of miR‐92a.

ROCK signalling pathway is one of the key regulators of cytoskeletal dynamics and determines the cell phenotypes, such as proliferation, migration, differentiation, and apoptosis, by reciprocal communication with the microenvironment.^45^ ROCK inhibitors have shown remarkable efficacy in reducing VSMCs contraction, endothelial dysfunction, inflammatory cell recruitment, vascular remodelling and cardiac remodelling.^46^, ^47^ MLCK and ROCK have been shown to phosphorylate both Thr‐18 and Ser‐19 of MLC to increase MLC phosphorylation status.^48^ Numerous cell activities, such as contraction, adhesion, cell migration and epithelial barrier formation occur in a MLC phosphorylation‐dependent or independent manner.^17^ Therefore, the ROCK and MLCK pathway are also known to be a key mediator of increased vascular reactivity.^49^, ^50^ As our above results indicated that the pathway of MLCK regulating miR‐92a is involved in the functional changes of VSMCs, it is necessary to further explore the relationship between ROCK and miR‐92a to clarify the mechanism of AS. Our results showed Y27632, a ROCK inhibitor, also inhibited miR‐92a expression and PDGF‐BB‐induced proliferation and migration of VSMCs. It is important to note that miR‐92a mimic partially rescued the effect of Y27632 on down‐regulation of VSMCs proliferation and migration. These results indicated that both ROCK and MLCK promoted the proliferation and migration of VSMCs through the positive regulation of miR‐92a.

MicroRNAs are processed from an initial stem‐loop structure of ~70 nucleotides by several dsRNA‐specific endonucleases and ultimately delivered as mature 20‐25 nucleotide species to RNA‐induced silencing complex where they engage in either translational arrest or degradation of targeted transcripts through imperfect base pairing with the 3 untranslated regions (UTR) of the targeted transcripts.^51^ miR‐92a inhibits KLF expression through 3’ UTR binding.^24^ KLF4 has been shown to act as potent repressor of VSMCs gene transcription in VSMCs through multiple mechanisms.^52^ We found ML‐7 to reduce lipid deposition lesions correlating with an increased KLF4 expression in aortic SMCs as KLF4 can suppress the proliferation and migration of VSMCs. Fang et al^24^ suggested that miR‐92a inhibitor‐mediated inhibition of TNF‐α‐induced cytokines and leucocyte adhesion can be rescued, in part, by siRNA‐KLF4, indicating partial KLF4 dependency. Our study also confirmed their finding. Indeed, miR‐92a inhibitor increased the KLF4 expression and miR‐92a mimic showed the opposite effect. Encouragingly, the inhibition of MLCK or ROCK enhanced the KLF4 expression. Above study suggest that KLF4 is down‐regulated by PDGF‐BB through MLCK/ROCK and miR‐92a. These results seem conflicting with the published findings^53^ and our Western blot experiments (Figure S6) that PDGF‐BB induced KLF4. For the relationship between PDGF‐BB and KLF4, we have the following considerations. PDGF‐BB, as a biochemical stimulator outside the cell, is located at the upstream of cell signalling, and its effects on cell functions, or transcription factors that regulate cell functions, are subject to complex network regulation in real time. Such as, PDGF‐BB‐induced increases in KLF4 expression were mediated through a Sp1‐dependent mechanism that involves the direct binding of Sp1 to the KLF4 promoter and requires three consensus Sp1 sites.^53^ But the signal pathway provided in this paper is only one of the signal pathways that PDGF‐BB regulates KLF4, that is the activation of ROCK and/or MLCK may up‐regulate miR‐92a expression, which subsequently inhibits the KLF4 expression and promotes PDGF‐BB‐mediated proliferation and migration of VSMCs.

The novelty of the current study rests on coupling the ROCK and MLCK pathways as well as finding the role of miR‐92a in AS. However, how are MLCK and ROCK as kinases involved in regulating the expression of miR‐92a? We know that the control of epigenetic regulation, transcriptional regulation, post‐transcriptional regulation and degradation level regulation, are the four major mechanisms of miRNAs expression.^54^, ^55^ A few papers showed that the transcription factor, STAT3, is involved in the regulation of miR‐92a expression. STAT3 can up‐regulate miR‐92a to inhibit PTEN or RECK target gene to promote cholangiocarinoma growth or lung cancer cells invasiveness.^58^, ^59^ Herein, we showed that ROCK/MLCK up‐regulated miR‐92a expression in VSMCs through STAT3 activation. Thus, the activation of ROCK/STAT3 or MLCK/STAT3 signalling may be an important factor for the induction of miR‐92a expression in VSMCs.

**Figure 8:**
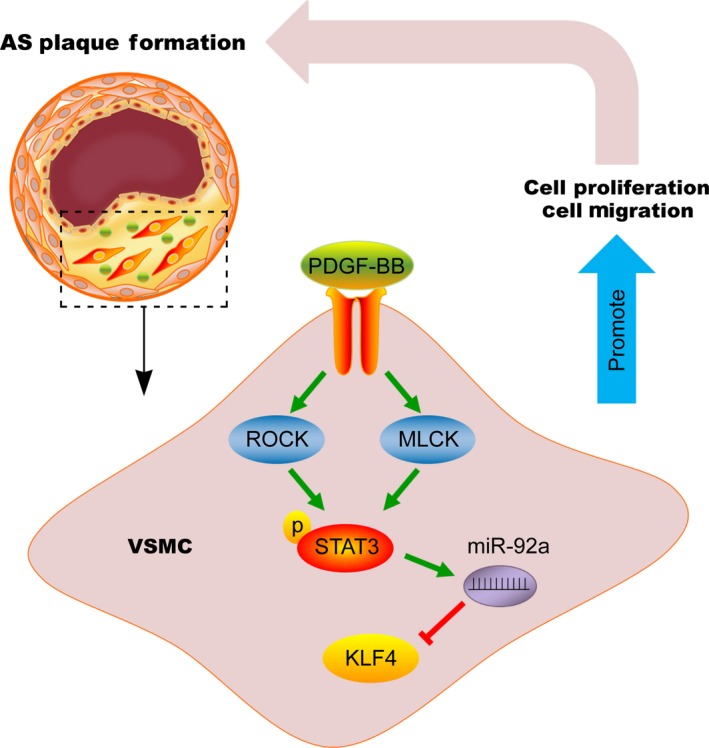
Schematic illustration of the proposed mechanism for the proliferation and migration of vascular smooth muscle cells (VSMCs). PDGF‐BB promotes the proliferation and migration of VSMCs through ROCK/STAT3 or MLCK/STAT3 up‐regulated microRNA‐92a (miR‐92a) which targets KLF4

In summary, we identified miR‐92a as a critical regulator in VSMCs proliferation and migration by targeting KLF4, at least partially. AS or PDGF‐BB is able to increase miR‐92a expression, indicating a pathological role of miR‐92a in proliferative vascular diseases. Increased miR‐92a expression could reduce the expression of its target gene *KLF4*. siRNA‐KLF4 increased the proliferation and migration of VSMCs. Moreover, both ROCK/STAT3 and MLCK/STAT3 were found to regulate miR‐92a expression. These findings suggested that ROCK and MLCK are upstream regulators for the expression of miR‐92a, which subsequently inhibits KLF4 expression and promotes PDGF‐BB‐mediated proliferation and migration of VSMCs (Figure 8). These discoveries on the known signalling pathways encourage a therapeutic approach for improving vascular functions of AS.

## CONFLICT OF INTEREST

All the authors confirm that there are no conflict of interest.

## AUTHOR CONTRIBUTIONS

J. Wang performed the experiments, analysed the data and wrote the paper; C. Zhang performed the experiments and analysed the data; C. Li performed the experiments. D. Zhao, S. Li, M. Li, Y. Cui and X. Wei analysed the data; L. Ma revised the paper; Y. Zhao designed and wrote the paper. Y. Gao designed and supervised research and revised the paper.
