# Supplementary figures and images for: MicroRNA‐92a promotes vascular smooth muscle cell proliferation and migration through the ROCK/MLCK signalling pathway
Source: J Cell Mol Med. 2019 Mar 25;23(5):3696–710. doi: 10.1111/jcmm.14274 (PMC6484312; doi:10.1111/jcmm.14274)

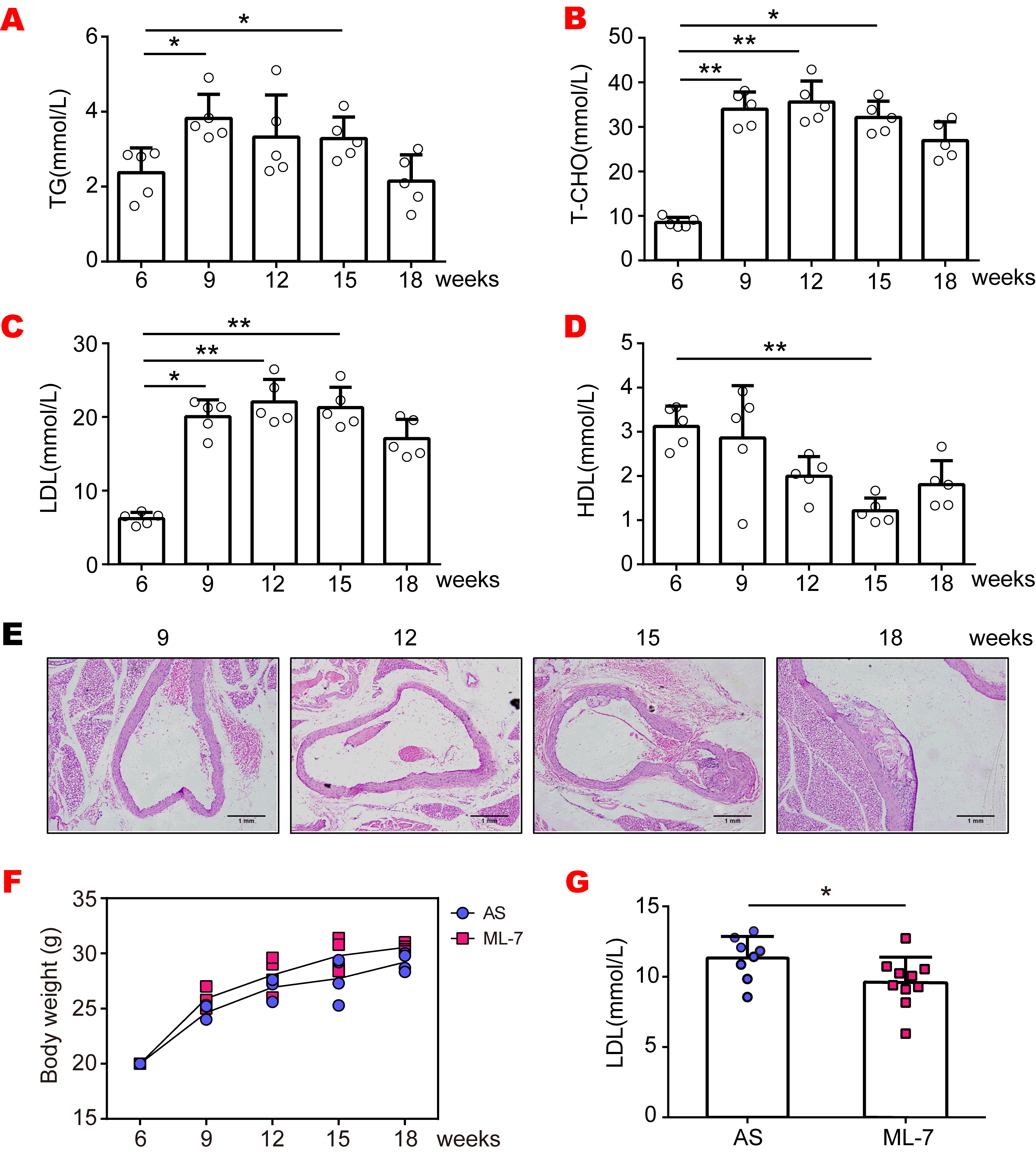

Supplement: Supplementary file 1 [file JCMM-23-3696-s001.tif]

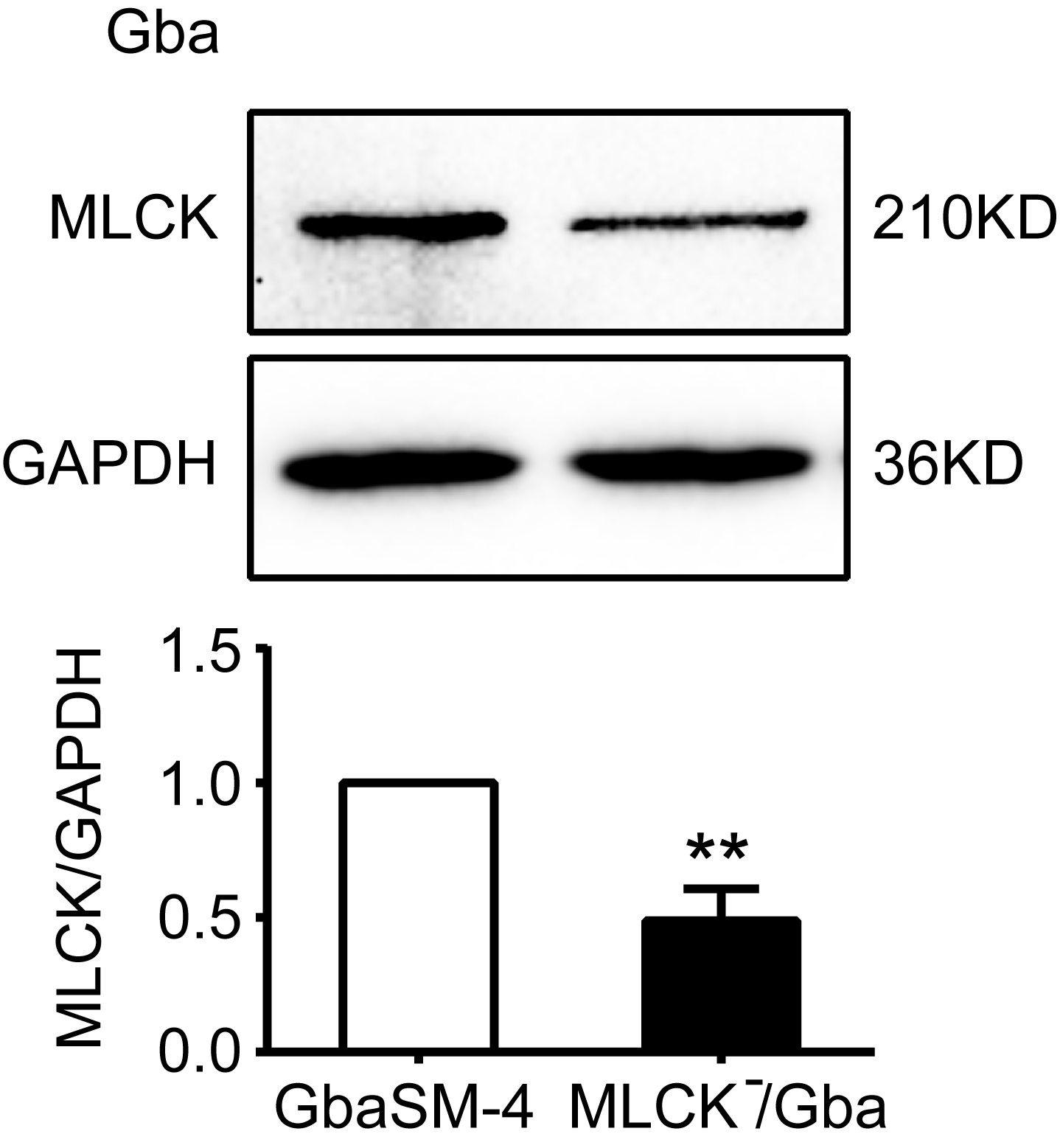

Supplement: Supplementary file 2 [file JCMM-23-3696-s002.tif]

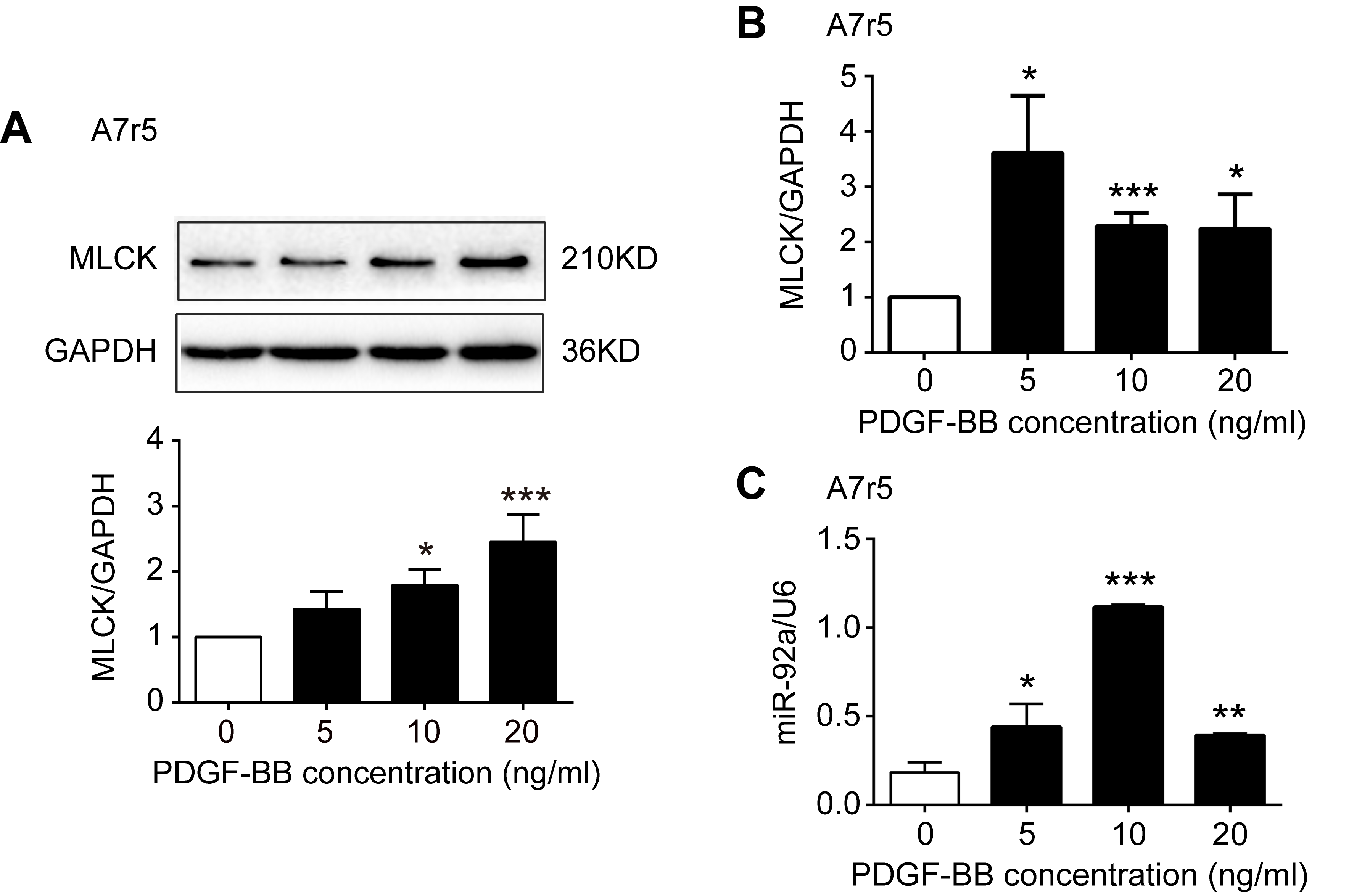

Supplement: Supplementary file 3 [file JCMM-23-3696-s003.tif]

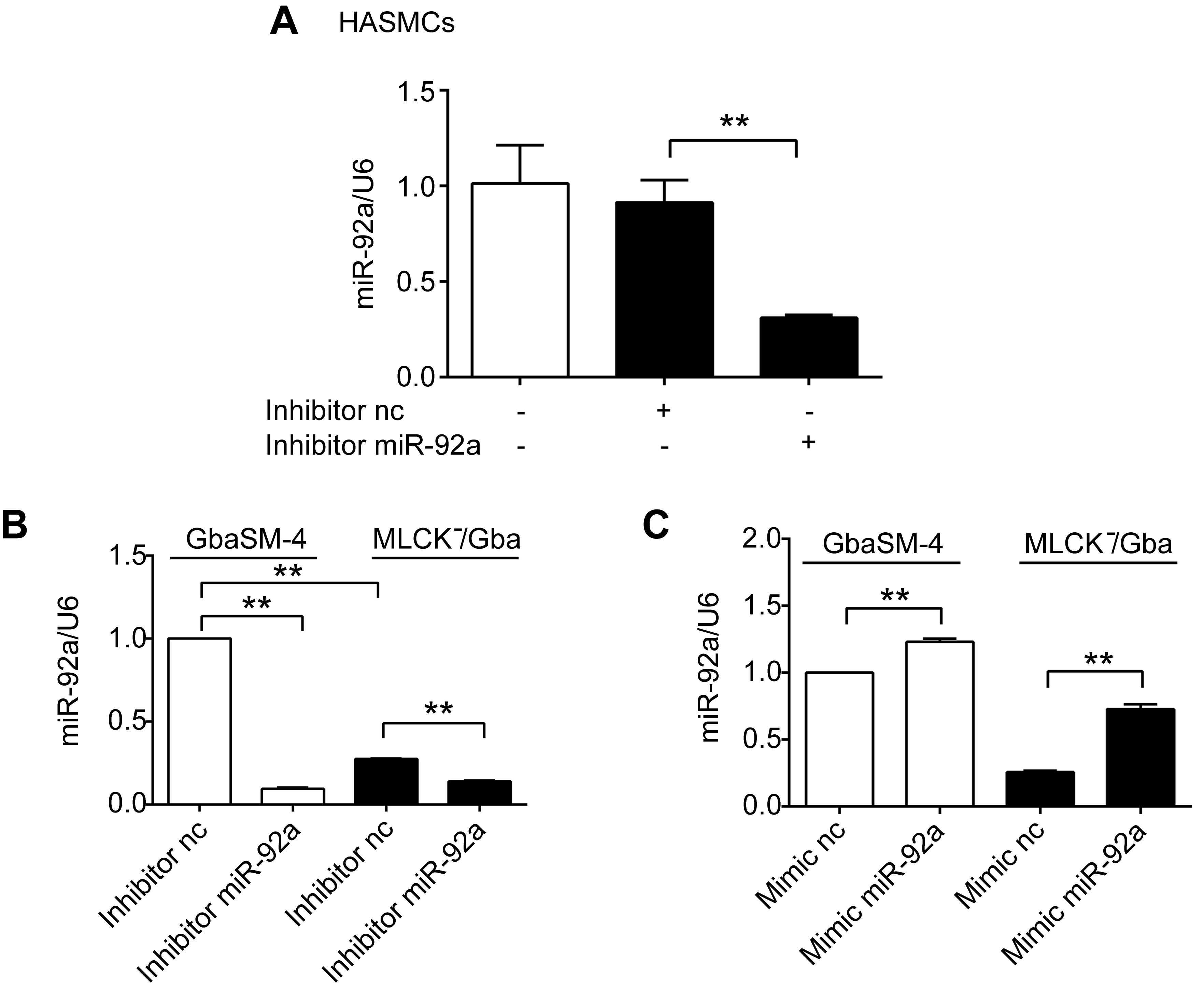

Supplement: Supplementary file 4 [file JCMM-23-3696-s004.tif]

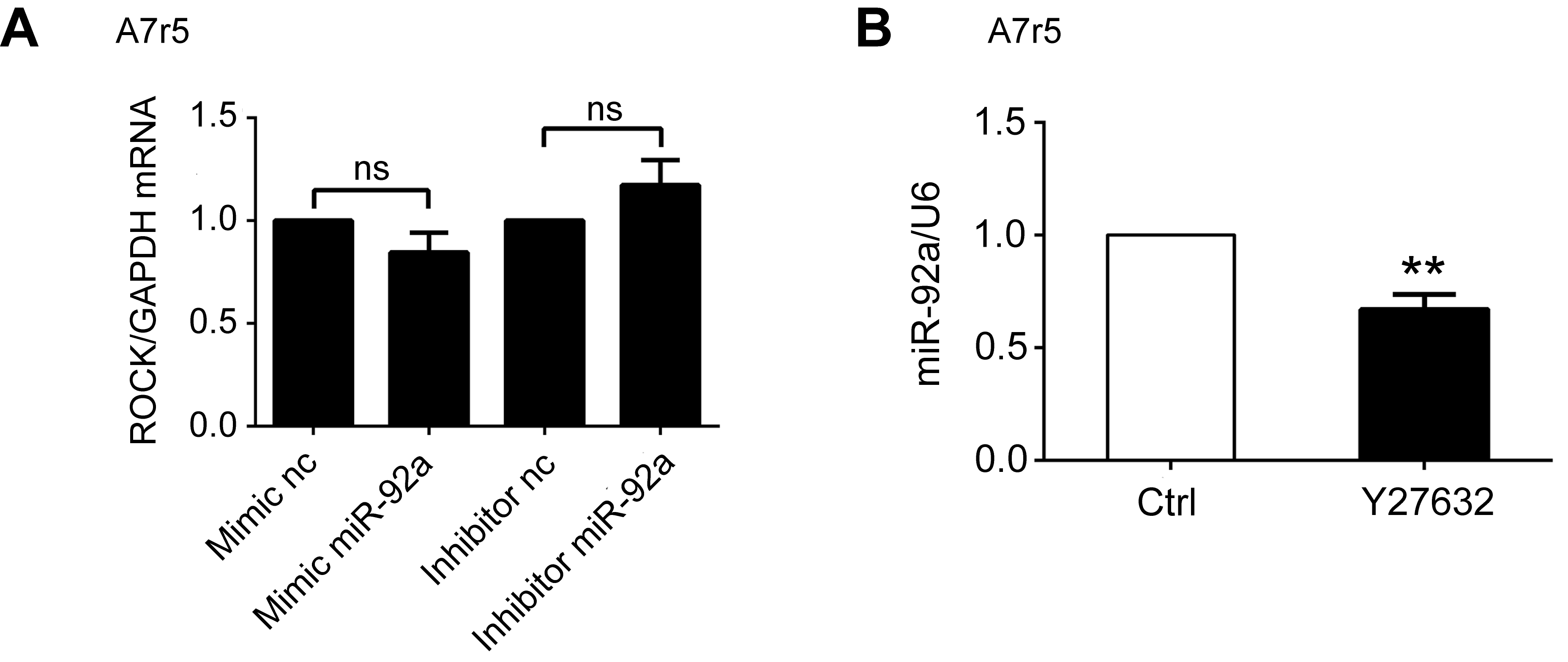

Supplement: Supplementary file 5 [file JCMM-23-3696-s005.tif]

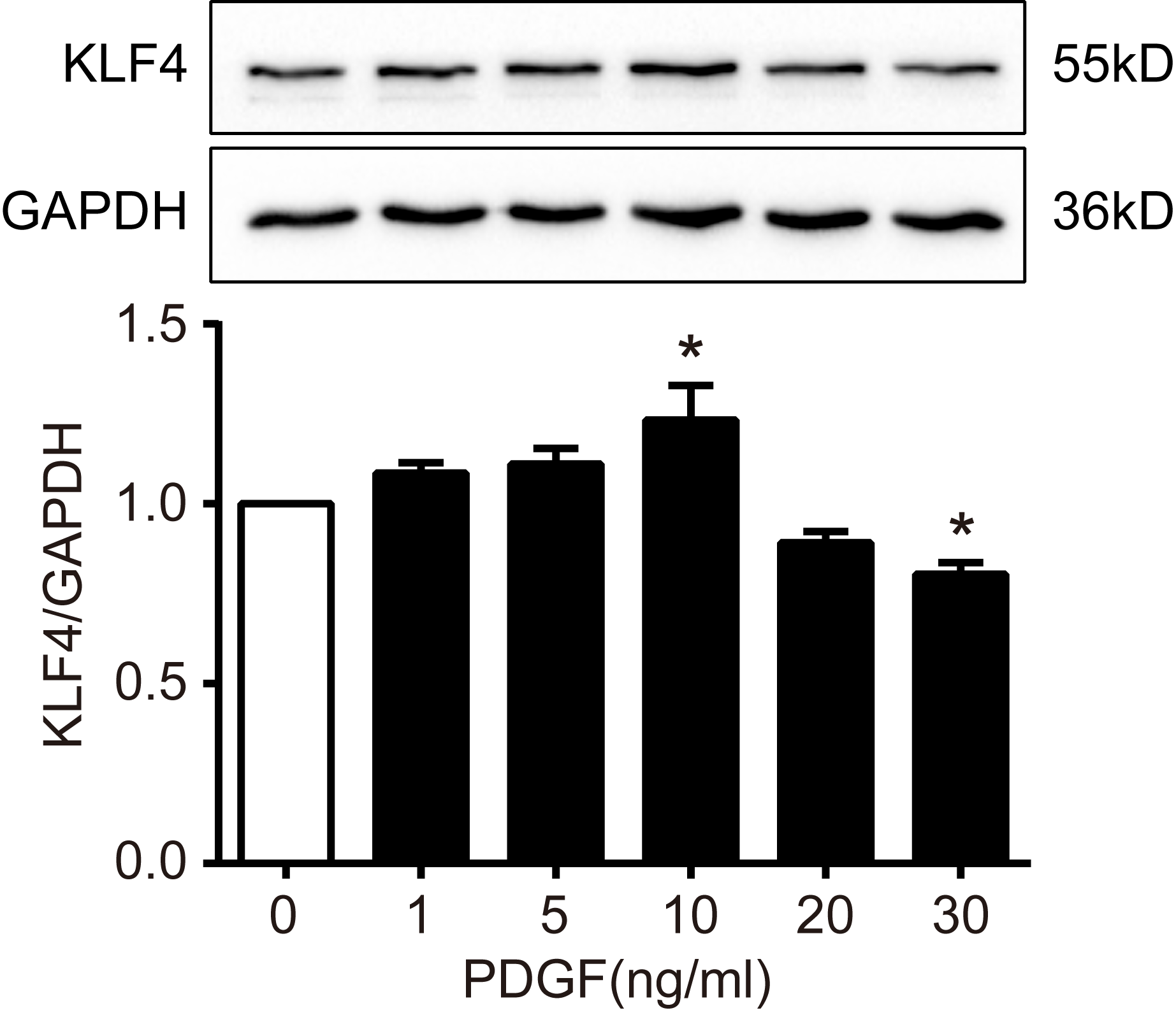

Supplement: Supplementary file 6 [file JCMM-23-3696-s006.tif]

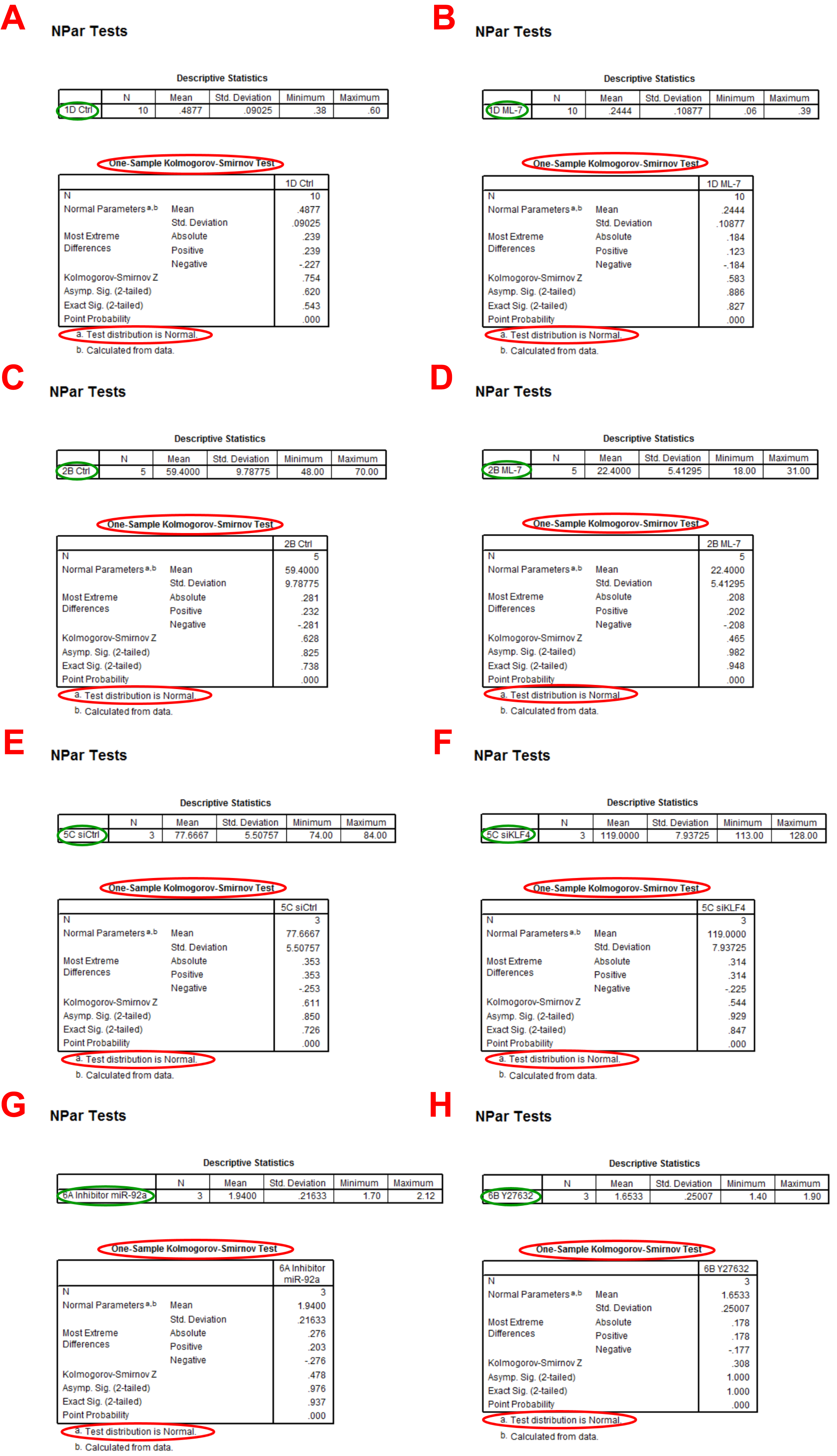

Supplement: Supplementary file 7 [file JCMM-23-3696-s007.tif]

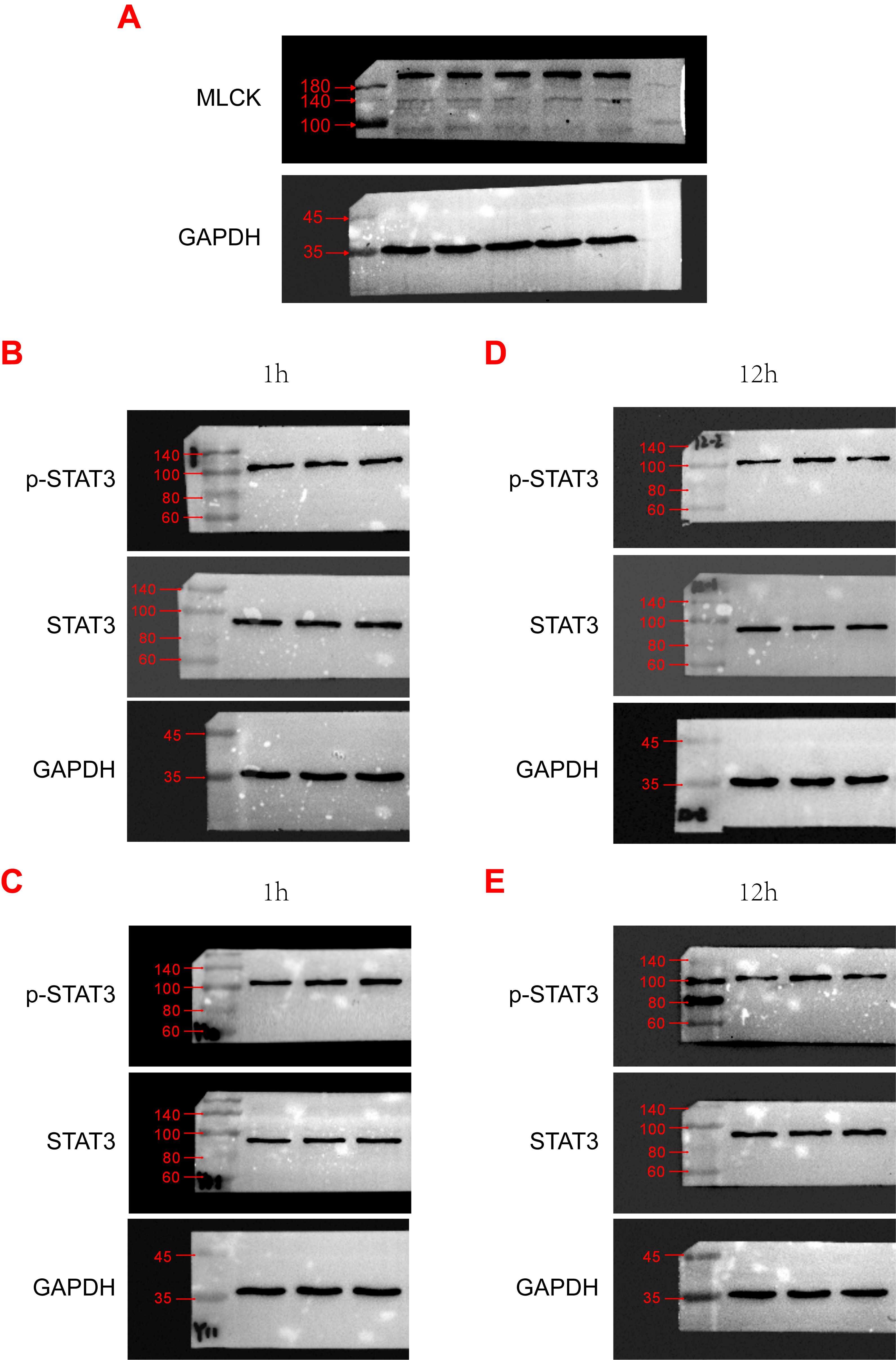

Supplement: Supplementary file 8 [file JCMM-23-3696-s008.tif]
