# Supplementary material for: MicroRNA‐92a promotes vascular smooth muscle cell proliferation and migration through the ROCK/MLCK signalling pathway
Source: J Cell Mol Med. 2019 Mar 25;23(5):3696–710. doi: 10.1111/jcmm.14274 (PMC6484312; doi:10.1111/jcmm.14274)
Supplement: Supplementary file 9 [file JCMM-23-3696-s009.docx]

**Supplemental Figure 1.** Establishment of AS mice model. (A-D) The serum was collected every 3 weeks from 6 weeks of age to measure the level of TG (A), T-CHO (B), LDL (C) and HDL (D). (E) Thoracic aorta structure of AS model group mice by HE staining (n=4). (F) The body weight of mice was measured (n=10). (G) The serum LDL level in ML-7 model group mice were measured. T-CHO, total cholesterol; TG, triglyceride; HDL, high-density lipoprotein; LDL, low-density lipoprotein. Data are presented as mean ± SD. **P< .*05, ***P< .*01.

**Supplemental Figure 2.** The expression of MLCK protein in GbaSM-4 and MLCK^-^/Gba cells. The protein level of MLCK were measured by Western blotting. Results of statistical analyses are shown below. Data are presented as mean ± SD. ***P< .*01.

**Supplemental Figure 3.** PDGF-BB enhance the expression of MLCK and miR-92a in A7r5. (A, B) The protein and mRNA level of MLCK were measured by Western blotting (A) and RT-qPCR (B) respectively in PDGF-BB-induced A7r5 cells. Results of statistical analyses are shown below. (C) miR-92a expression levels were measured by RT-qPCR in PDGF-BB-induced A7r5 cells. Data are presented as mean ± SD. **P< .*05, ***P< .*01, ****P< .*0001.

**Supplemental Figure 4.** The expression of miR-92a changed by transfection of miR-92a inhibitor and miR-92a mimic in VSMCs. (A) miR-92a expression levels was assessed by RT-qPCR after transfected. (B, C) miR-92a expression levels in GbaSM-4 and MLCK^-^/Gba cells was assessed by RT-qPCR after transfection with miR-92a inhibitor (B) or miR-92a mimic (C). Data are presented as mean ± SD. ***P< .*01.

**Supplemental Figure 5.** The relationship between ROCK and miR-92a in A7r5. (A) The mRNA levels of ROCK were measured by RT-qPCR. (B) miR-92a expression levels were assessed by RT-qPCR in A7r5 cells treated with ML-7 (10 µM) for 1 h. ns, no significant; nc, negative control. Data are presented as mean ± SD. ***P< .*01.

**Supplemental Figure 6.** The relationship between PDGF-BB and KLF4 in HASMCs. HASMCs were treated with different concentrations of PDGF-BB. The protein levels of KLF4 were measured by Western blotting. Data are presented as mean ± SD. **P< .*05.

**Supplemental Figure 7.** The K-S test was used to check the data distribution before using the Student's t test for statistical analysis. (A, B) The results of K-S Test for control group (A) or ML-7 treatment group (B) of Figure 2B. (C, D) The results of K-S Test for control group (C) and ML-7 treatment group (D) of Figure 2B. (E, F) The results of K-S Test for siCtrl group (E) and siKLF4 group (F) of Figure 5C. (G) The results of K-S Test for inhibitor miR-92a group of Figure 6A. (H) The results of K-S Test for Y27632 group of Figure 6B.

**Supplemental Figure 8.** The original Western blotting bands with merged markers (A) Original images of Figure 1H. (B-E) Original images of Figure 7C-F.
